# Hippocampal-Dependent Cognitive Dysfunction following Repeated Diffuse Rotational Brain Injury in Male and Female Mice

**DOI:** 10.1089/neu.2021.0025

**Published:** 2021-05-13

**Authors:** Laura B. Tucker, Amanda H. Fu, Joseph T. McCabe

**Affiliations:** ^1^ Center for Neuroscience and Regenerative Medicine, Physiology and Genetics, Uniformed Services University of the Health Sciences, Bethesda, Maryland, USA.; ^2^ Department of Anatomy, Physiology and Genetics, Uniformed Services University of the Health Sciences, Bethesda, Maryland, USA.

**Keywords:** fear conditioning, hippocampus, learning, memory, Morris water maze, sex differences

## Abstract

Cognitive dysfunction is a common, often long-term complaint following acquired traumatic brain injury (TBI). Cognitive deficits suggest dysfunction in hippocampal circuits. The goal of the studies described here is to phenotype in both male and female mice the hippocampal-dependent learning and memory deficits resulting from TBI sustained by the Closed-Head Impact Model of Engineered Rotational Acceleration (CHIMERA) device—a model that delivers both a contact–concussion injury as well as unrestrained rotational head movement. Mice sustained either sham procedures or four injuries (0.7 J, 24-h intervals). Spatial learning and memory skills assessed in the Morris water maze (MWM) approximately 3 weeks following injuries were significantly impaired by brain injuries; however, slower swimming speeds and poor performance on visible platform trials suggest that measurement of cognitive impairment with this test is confounded by injury-induced motor and/or visual impairments. A separate experiment confirmed hippocampal-dependent cognitive deficits with trace fear conditioning (TFC), a behavioral test less dependent on motor and visual function. Male mice had greater injury-induced deficits on both the MWM and TFC tests than female mice. Pathologically, the injury was characterized by white matter damage as observed by silver staining and glial fibrillary acidic protein (astrogliosis) in the optic tracts, with milder damage seen in the corpus callosum, and fimbria and brainstem (cerebral peduncles) of some animals. No changes in the density of GABAergic parvalbumin-expressing cells in the hippocampus, amygdala, or parietal cortex were found. This experiment confirmed significant sexually dimorphic cognitive impairments following a repeated, diffuse brain injury.

## Introduction

Traumatic brain injury (TBI) is a growing worldwide health burden, increasing in prevalence by 8.4% between 1990 and 2016.^1^ Although the majority of sustained TBI cases are mild (mTBI), the functional consequences affect millions of individuals worldwide and include motor, cognitive, and psychiatric symptoms.^2^ These issues are associated with significant disability^3^ and may affect patients for over 10 years.^4,5^ Many patients require long-term care, placing significant burden on health-care systems and caretakers. In recent years, there also has been increasing attention directed toward the effects of repeated mTBI, often sustained by both men and women in sports and military contexts.^6,7^ Repeated mTBI places individuals at greater risk for long-term symptoms, and is also associated with neurodegenerative conditions such as chronic traumatic encephalopathy.^8,9^

Unfortunately, little is known about the biological mechanisms underlying neurobehavioral symptoms following mTBI, although axonal injury is a common finding with imaging methods following concussion.^10,11^ Animal models of TBI have provided a controlled environment in which the effects of injury can be manipulated and studied, and white matter damage has been demonstrated in rodent models of single and repeated mTBI.^12–16^ Behavioral dysfunction, including hippocampal-dependent learning and memory deficits, are also reliably observed following TBI in rodents^17,18^ and several drugs have been identified in translational studies that prevent or reverse both axonal damage and cognitive deficits.^15,19-21^ However, despite the numerous promising pharmacological agents recognized in pre-clinical trials, all phase III clinical trials have failed and at present there is no U.S. Food and Drug Administration–approved therapy for TBI.^22,23^ Nevertheless, translational models of clinical relevance remain critical in furthering the understanding of the pathophysiological cascades following injury, functional consequences of TBI, and subsequent testing of potential therapeutic agents.

There are numerous rodent models of TBI available to investigators.^24–27^ Some of the most popular models for the past few decades are relatively invasive, requiring craniectomies, such as controlled cortical impact (CCI)^28^ and fluid percussion injury (FPI).^29^ CCI and FPI result in focal and combined focal-diffuse injuries, respectively, and are valuable for the study of local biological responses to contusional forces.^24^ Although these methods remain popular, there has been growing interest in models that are milder and considered more clinically relevant, directing the impact to the scalp or skull rather than the dura mater, but still keeping the head fixed in place to more precisely control impact conditions. However, early pioneering work employing subhuman primates demonstrated that free movement of the head, generating acceleration and deceleration forces rather than contact forces, was critical in generating the ensuing pathological responses,^30-32^ and there have been some several modern rodent models where impact has allowed for head displacement and subsequent unrestrained head movement.^33–36^

The Closed-Head Impact Model of Engineered Rotational Acceleration (CHIMERA) model of experimental brain injury is a relatively recently developed, commercially available model that incorporates aspects of contact-concussion with acceleration/deceleration and rotational injury. CHIMERA was introduced approximately 7 years ago^14^ and has since been demonstrated in approximately 20 publications to date to reliably elicit diffuse white matter injury and cognitive deficits in rodents.^37^ Learning and memory deficits after CHIMERA injuries, single or repeated, have been shown with the Morris water maze (MWM), Barnes maze, and passive avoidance behavioral tests in mice,^37^ all of which depend on intact sensory and motor function during test performance. Recently, Desai and colleagues showed that following three CHIMERA injuries, male mice were impaired on a visible platform test in the MWM and on the visual cliff test of visual acuity, and also had reduced visual evoked potentials.^38^ These findings were coupled with increased inflammation in the optic tracts, a common finding following single or repeated mTBI in rodent models.^39–42^ This behavioral, physiological and pathological evidence suggests that brain-injured mice suffer visual dysfunction, which may interfere with behavioral testing.

The goal of the current studies is to describe learning and memory deficits in the MWM following multiple CHIMERA injuries in both male and female mice. In addition, the findings in the MWM are supplemented with data from trace fear conditioning (TFC), another hippocampal-dependent behavioral test that is less dependent on sensory and motor function. We hypothesize that although sensory and motor deficits may be apparent during MWM testing and cloud conclusions regarding learning and memory function, TFC testing will support conclusions of cognitive dysfunction. Further, it is hypothesized that male mice will have greater deficits on cognitive tests than female mice following injury, as previous studies have demonstrated such differences.^13,39,43^

## Methods

### Animals and housing

All animal procedures were approved by the Institutional Animal Care and Use Committee at the Uniformed Services University of the Health Sciences (USUHS; Bethesda, MD). Male and female mice, 8 weeks old, were obtained from Jackson Laboratories (C57BL/6J, 000664; Bar Harbor, Maine) and group-housed (4-5 per cage) in Association for Assessment and Accreditation of Laboratory Animal Care–accredited facilities with a standard 12-h light–dark cycle, with food (Harlan Teklad Global Diets 2018, 18% protein) and water available *ad libitum.* Animals acclimated to facilities for 7-10 days prior to baseline behavioral testing. All procedures involving rodent handling were performed by female investigators.^44^

### CHIMERA procedures

Mice were randomly assigned to sustain sham procedures or four CHIMERA brain injuries, delivered at 24-h intervals. CHIMERA procedures were performed as initially described by Namjoshi and colleagues.^14^ Mice were anesthetized with isoflurane (3% in 100% oxygen) in a clear induction chamber; anesthesia (2.5%) was maintained via nosecone while the animal was positioned on the device. The animal was mounted in a supine position in the animal holder, with the head flat over a hole in the head plate and the body angled approximately 32°. Crosshairs across the piston hole aid in aligning the animal's head, resulting in reliable impact to the dorsal cortical region. The body was held in place on the platform with Velcro straps, allowing the head to rotate in the sagittal plane when the piston was deployed, resulting in an impact rotational injury. Instrument pressure was set at 3.8-4.46 psi to obtain a piston velocity of 5.29 m/sec, which corresponds to an energy level of 0.7 J. (Energy (J) = (1/2m) × v^2^, where m is the mass of the piston (0.05 kg) and v is the piston velocity.) Actual measured velocity for all impacts had an average of 5.2762, resulting in calculated energy of 0.6960 J with 2.36% coefficient of variation. Sham-treated mice underwent all procedures, including anesthesia and positioning on the device, but the impact was not delivered. The total duration of isoflurane exposure each day was approximately 4.5 min for both injured and sham-control mice.

Any occurrence of apnea immediately following the impact was noted, measured, and recorded. Injured and sham-treated mice were placed in a clean cage in a supine position immediately after cessation of anesthesia, and the latency to return to a prone position was recorded as the righting reflex. All mice received acetaminophen in their drinking water beginning when they were returned to their home cages after the first procedure, continuing until 24 h following the final injury or sham procedure (1 mg/mL; approximately 200 mg/kg).

### Behavioral testing

#### Experiment 1—Spontaneous activity, motor and cognitive behavior

A total of 59 mice were included in Experiment 1: Male Chimera, 15; Male Sham, 15; Female Chimera, 14; and Female Sham, 15. Open field (OF), rotarod, y-maze (spontaneous alternation test, working memory) and Morris water maze (MWM, spatial learning and memory) testing was performed as previously described.^45,46^ Body weights were measured weekly. Baseline OF measurements were taken approximately 4 days prior to injury procedures, and OF behavior was assessed on Days 1, 7, 14, and 21 post-injury. The OF apparatus (Stoelting Co.) was a 40 cm × 40 cm arena with opaque black walls approximately 40 cm high (∼ 5 Lux). Each arena had an overhead camera connected to a computer with Any-Maze software (Stoelting Co.) that tracked movements of the mice during a 20-min testing session. Measures recorded included total distance traveled, time spent in an immobile state (defined as 70% of the animal remaining motionless for at least 2 sec), and movement speed while mobile. One female sham mouse was excluded from all OF analysis due to abnormally high spontaneous activity levels (total distance traveled) during baseline testing (9.0 standard deviations [SD] above the group mean).

Mice were trained to perform on an accelerating rotarod (4-60 rotations/min over 3 min) for 3 days prior to injury.^46^ The latency to fall from the rod or the time at which the mouse clung to the rod for three consecutive rotations was recorded and averaged for three trials each day. Performance on the 3rd training day was recorded as the baseline value, and post-injury behavior was assessed on Days 1, 7, 14, and 21. Rotarod testing on post-injury days was performed following open field assessment.

Hippocampal-dependent working memory was assessed on Day 10 following CHIMERA injuries by testing spontaneous alternation behavior in the y-maze.^45^ The apparatus (Stoelting, CO) consisted of three arms (36 cm long with 16 cm high walls) at a 120° angle to one another, meeting at a central triangular zone. Testing was performed at approximately 15 Lux. Mice were individually placed at the end of a randomly chosen arm, and the mouse was free to explore all arms during a 5-min testing session. Objects around the room provided spatial cues. Movements of the mice were recorded by an overhead camera and entries to the arms were later scored by an observer blinded to the injury condition of the animal. A mouse with intact working memory is expected to display spontaneous alternation behavior, visiting all three arms in alternation, not returning to either of the arms most recently explored. An alternation was counted when the mouse entered the three arms consecutively, and percent correct alternation was calculated as 100 × $\frac{totalnumberofalternations}{totalarmentries-2}$ .

Hippocampal-dependent spatial learning and memory was tested in the Morris water maze (MWM) on Days 24-37 following CHIMERA injuries.^45^ Spatial learning trials were conducted for 4 days on Days 24-27 following injuries with a probe trial conducted 24 h later (Day 28). Four days of reversal learning trials began on Day 31, and 24 h following completion a reversal probe trial was conducted. Finally, 2 days later (37 days following injuries), mice underwent four visible platform trials. The MWM apparatus was a white circular tank (122 cm diameter) filled with water (21 ± 1°C) to a depth of about 30 cm. A round transparent platform (11 cm diameter) was submerged just below the surface of the water approximately 15 cm from the edge of the maze. Diffuse room lighting provided illumination of approximately 60 Lux. An overhead camera connected to a computer with Any-Maze software recorded movements of the mice during testing; within the software, the circular maze was divided into four equal quadrants.

Four trials were performed on each learning day, with an inter-trial interval of 3-4 min. The animal was placed, facing the wall of the maze, at a different start position along the perimeter of the tank for each trial, and the order of the start positions was varied each day. Mice were allowed 60 sec to find the hidden platform using spatial cues (large black and white geometric shapes on the walls), after which it remained on the platform for 15 sec. If the mouse did not find the platform in the allotted time, it was gently guided to the platform, allowed to remain there for 15 sec, and assigned 60 sec as the latency score for that trial. Following each trial, mice were gently removed from the maze, towel-dried, and placed into a heated cage. The software recorded the latency to find the platform, distance swam to the platform, and the swimming speed, and these measures were averaged over the four trials each training day.

The day after the final training trials (Day 28 following injuries) a single probe trial was conducted in which the platform was removed from the maze. The mouse was placed in the maze directly opposite the location in which the platform had been formerly located, facing the wall, and allowed a 60-sec swim. The software recorded the time in which the animal spent in the northwest (NW) quadrant (that previously housed the platform) and the number of times the mouse crossed the previous exact location of the platform.

Beginning on Day 31 post-injury (3 days following the probe trial), reversal learning trials began. These trials were identical to the original spatial training trials on Days 24-27, except the hidden platform was moved to the opposite (southeast; SE) quadrant. The day following completion of reversal training trials (post-injury day 35), a reversal probe trial in which the platform was removed from the maze was performed.

Four visible platform trials were conducted two days following the reversal probe trial (Day 37 following injury). A highly visible (patterned) flag was secured to the platform, placed in the center of the maze, and each mouse underwent four trials. A different start position was employed for each trial, and the software recorded the latency to find the platform and the distance swam to the platform.

#### Experiment 2—Trace fear conditioning

The TFC paradigm was employed in an additional 69 mice as a separate assessment of hippocampal-dependent learning.^47–50^ Mice (Male CHIMERA, 16; Male Sham,14; Female CHIMERA, 22; Female Sham, 17) were tested on Days 24-26 following CHIMERA or sham procedures. During conditioning on the first day, mice were placed in Plexiglas fear conditioning chambers (17 cm × 17 cm, ∼5 lux; Ugo-Basile, Varise, Italy) with a metal rod floor, within sound-attenuating and light-tight cubicles. Salient black and white checkerboard or striped walls provided visual cues, and odor cues were provided with mint or lemon extract. Following a 3-min acclimation period, the animals were presented with a 70 dB white noise conditioned stimulus (CS), 20 sec in duration. After a 20 sec delay, the auditory stimulus was followed by a foot-shock, 0.6 mA and 2 sec in duration. The noise-shock pairing was repeated three more times, with intervals between the shock and next CS 210 sec, 150 sec, and 270 sec, for a total of four pairings. The animals remained in the chambers for 1 min following the final shock. To reduce stress for un-tested animals, mice were placed into holding cages following conditioning until all mice from an individual housing cage had completed testing.

The tone test was performed the day following conditioning. The chamber was altered with modified visual, light, tactile and odor cues. After a 3-min baseline, mice were presented with the 20-sec white noise CS (70 dB), followed by an inter-tone interval of 20 sec. The CS was repeated four more times, for a total of five presentations of the noise and inter-trial interval. The amount of time mice spent freezing was recorded by Any-Maze software (Minimum freeze duration: 250 msec; Freezing on threshold: 30 (no units); Freezing off threshold: 40 [no units]). Freezing detection is performed by Any-Maze by analyzing movement throughout the apparatus, taking into account that noise will be present, such as flickering of individual video pixels and breathing of the animal.

On the 3rd day, the contextual exposure test was performed with contextual conditions in the testing chamber identical to what they were during conditioning on the 1st day. Mice were placed in the chamber, and the amount of time spent freezing during a 5-min test session was recorded by Any-Maze software.

### Histology and immunohistochemistry

Following behavioral testing, 37 days following the final injury, mice were deeply anesthetized (60 mg/kg ketamine, 60 mg/kg xylazine, intraperitoneally) and transcardially perfused with 0.1 M phosphate buffer (PB) followed by 4% paraformaldehyde (PFA) in 0.1 M PB. Brains were removed and post-fixed overnight (4% PFA in 0.1 M PB), then transferred to 20% sucrose (in 0.1 M phosphate buffer) for cryoprotection. Following at least 24 h of cryoprotection, brains were frozen in dry ice powder and stored at -80°C.

Six brains from each injury and sex group (three from Experiment 1 and three from Experiment 2) were randomly selected and processed for hematoxylin and eosin (H&E), glial fibrillary acidic protein (GFAP), and parvalbumin staining by FD NeuroTechnologies, Inc. (FDN; Columbia, MD). Serial cryostat sections (30 μm) were taken coronally from approximately bregma -0.94 to -4.16 mm.^51^ Sets of sections at 210-μm intervals were processed separately for H&E, GFAP and parvalbumin. The first set of sections was mounted on Superfrost Plus microscope slides (Thermo Scientific, Portsmouth, NH), and stained with FD H&E solution^TM^ (FDN). The second and third sets were processed for GFAP and parvalbumin-immunoreactivity, respectively.

After inactivating endogenous peroxidase activity with 0.6% H_2_O_2_, sections were incubated free-floating at 4°C for 43 h in 0.01 M phosphate-buffered saline (PBS, pH 7.4) with 1% normal donkey serum (Jackson ImmunoResearch, West Grove, PA), 0.3% Triton X-100 (Sigma, St. Louis, MO) and either rat monoclonal anti-GFAP immunoglobulin G (IgG; 1:20,000; Cat. #: 130300, Invitrogen, Carlsbad, CA) or rabbit polyclonal anti-parvalbumin IgG (1:20,000; Cat. #: ab11427, Abcam, Cambridge). The immunoreaction product was then visualized with the Vectastain Elite^®^ ABC kit (Vector Lab., Burlingame, CA) according to the avidin-biotin complex method of Hsu and colleagues.^52^ Briefly, sections were incubated in PBS-containing biotinylated goat anti-rat (for GFAP sections) or anti-rabbit (for parvalbumin sections) IgG, normal goat serum and Triton-X for 1 h and then in PBS containing avidin-biotinylated horseradish peroxidase complex for 1 h, followed by incubation of the sections in 0.05 M Tris buffer (pH 7.2) containing 0.03% 3′,3′-diaminobenzidine (Sigma) and 0.0075% H_2_O_2_ for 5 min. After thorough rinsing in distilled H_2_O, sections were mounted on slides, dehydrated in ethanol, cleared in xylene, and cover-slipped in Permount^®^ (Fisher Scientific, Fair Lawn, NJ). Unless otherwise noted, all steps were performed at room temperature and followed by washes in PBS.

An additional 5-6 brains from each injury and sex group (approximately equal numbers from Experiments 1 and 2) were processed at USUHS for silver staining. Brains were sectioned (30 μm) with a sliding microtome and sections were stored at -20°C in cryoprotectant until processing. Following seven days of incubation in PFA (4%) in 0.1M PB at 4°C, silver staining was performed employing FD NeuroSilver^TM^ Kit II, following the manufacturer's instructions.

All slides were scanned with a Zeiss AxioScan Z1. Zen 2.5 software (blue edition, ^©^Carl Zeiss Microscopy) was employed to capture regions of interest (ROIs) from sections beginning at approximately bregma -1.50 (Franklin & Paxinos): GFAP: bilateral parietal cortex (pCTX), corpus callosum (CC), bilateral optic tracts (OTs), bilateral hippocampus (HP), bilateral fimbria of the hippocampus (FI); parvalbumin (pCTX, HP, bilateral amygdala (AMY); silver stain (CC, FI, OT, cerebral peduncles (CP; Supplementary Fig. S1). H&E and additional GFAP and parvalbumin sections were analyzed from somatosensory and motor cortex (smCTX; from close to the impact site, beginning at approximately bregma -0.70). Images of ROIs for GFAP and parvalbumin-stained sections were imported into ImageJ software for further processing. pCTX and smCTX were analyzed in 500 μm × 500 μm regions, 1000 μm from midline; the CC, OTs, and FIs were analyzed in 800 μm × 600 μm, 350 μm × 350 μm, and 250 μm × 250 μm regions, respectively.

The percent area stained of each ROI was determined for GFAP-stained black/white images employing the threshold feature after the ROI was manually selected, and the ratio of area of positive signal to the total area is expressed as the percent area stained. Parvalbumin-stained images were analyzed similar to methods previously described.^39^ The particle analysis feature of ImageJ was employed to count cells on thresholded, black/white samples. Values for GFAP- and parvalbumin-analyzed slides were typically averaged across three sections per animal, approximately 210 μm apart.

Silver- and H&E-stained images are described qualitatively. All image analyses were performed by an investigator blinded to all experimental conditions of the animals from which the sections were taken.

### Statistical analysis

Statistical analyses were performed with SAS Studio 3.8 (SAS Institute Inc., Cary, NC, USA) and SPSS (version 21; IBM SPSS Statistics, Armonk, NY). Righting reflexes were analyzed by Kruskal-Wallis tests performed for each injury day followed by Dunn-Bonferroni-corrected *post hoc* multiple comparisons (SPSS). Behavioral measures recorded at multiple time-points (OF, rotarod, MWM training data) were analyzed in mixed models with Injury and Sex as fixed factors and Day as a repeated measure. Data from standard and reversal trials in the MWM were analyzed separately. Latency and distance traveled to platform from visible platform trials in the Morris water maze did not meet homogeneity of variance requirements as assessed with Levene's homogeneity of variance test; these values were transformed to natural log values prior to analyses. Two-way analyses of variance (ANOVAs; Injury × Sex) were performed for freezing during TFC for the baseline periods, and for the total amounts of freezing during tone and trace periods separately. Immunohistological (GFAP and parvalbumin) data were analyzed with ANOVAs with Sex and Injury as fixed factors and Side (e.g., AMY, OT) and/or Subregion (i.e., HP) as repeated measures). Interaction effects were followed up by Bonferroni-corrected planned contrasts (*t*-tests). Where appropriate, following significant main effects or planned contrasts, Cohen's *d* effect size was calculated as $\left|\frac{\mu_{1}-\mu_{2}}{s_{pooled}}\right|$, where s_pooled_ = $\sqrt{\frac{s_{1}^{2}+s_{2}^{2}}{2}}$ .

Figures were designed with Microsoft Excel 2016 and Daniel's Excel XL Toolbox 7.34. Unless indicated otherwise, data presented in figures represent the mean ± standard error of the mean.

## Results

### Mortality, apnea, and righting reflexes

Mortality rate as a result of CHIMERA procedures was approximately 7.5%. There were 72 injured mice originally included in the study (Experiments 1 and 2); five of those mice died or were humanely euthanized following injury procedures. One of the mice was a female mouse that was humanely euthanized following the second injury day. The other four mice were male mice; the mice died following injury procedures. One of the male mice died following the first injury, one following the third, and the other two mice died following the final CHIMERA injury.

**FIG. 1.**
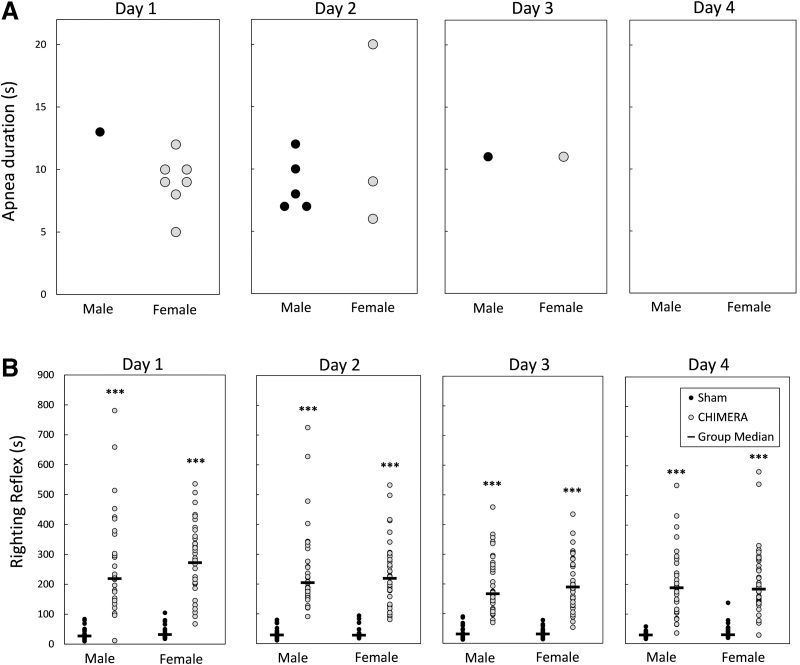
Apnea **(A)** and recovery of righting reflexes **(B)** following Closed-Head Impact Model of Engineered Rotational Acceleration (CHIMERA) injury or sham treatment each day. Legend in (B) applies to both panels. Represented in (A) are specific instances of apnea each day of injury. There were very few incidences of apnea following injury (none after sham procedures), and they tended to occur after the earlier injuries. The righting reflex (time to return to a prone position after being placed supine following discontinuation of anesthesia) is shown in (B). On all injury days, both male and female injured mice took significantly longer to right themselves than their sex-matched sham-treated mice (***p < 0.001 for all days, CHIMERA > Sham).

Apnea was rarely observed following CHIMERA procedures. CHIMERA procedures were performed 268 times: Experiment 1 had 29 injured mice, injured 4 × each for a total of 116 CHIMERA procedures and Experiment 2 had 38 injured mice totaling 152 procedures. Of the 268 CHIMERA procedures, apneic episodes occurred following 15 injuries (∼5%). Figure 1A shows the occurrence and durations of apnea following injury each day. Apnea was observed in six mice on injury Day 1 (one male and five females), seven mice on Day 2 (four males and three females), and two mice on Day 3 (one male and one female). No mice were observed to have apnea on the final day of injury.

Figure 1B shows the latency to regain the righting reflex following injury procedures for all mice in Experiments 1 and 2. Kruskal-Wallis tests performed for each injury day showed that both male and female injured mice had longer latencies to right themselves following injury than sham controls of the same sex on all injury days [H(3) = 89.52, 94.94, 93.51, 88.99 for Days 1-4, respectively; *p* < 0.0001]. There were no statistical sex differences in the injured or sham-control groups on any of the injury days (*p* = 1.0).

### Body weights

Supplementary Figure S2 shows body weights for mice in Experiment 1 on Days 1, 7, 14, 21, 28, and 35 post-injury. There was a significant Injury × Day interaction effect on the percent weight change following injury (F_5,51_ = 4.49, *p* = 0.0018). Planned contrasts comparing injured and sham-treated mice on each post-injury day showed that injured mice had reduced body weights compared with sham controls on post-injury Days 7 (*p* < 0.0001, *d* = 1.25), 14 (*p* < 0.0001, *d* = 1.45), 28 (*p* < 0.0001, *d* = 1.38), and 35 (*p* < 0.0001, *d* = 1.34). There were no main effects of Sex, or interactions between Sex and Injury or Day (F ≤ 0.98, *p* ≥ 0.4393).

### Pathological findings

#### H&E

Qualitative analysis of H&E-stained sections did not reveal any gross evidence of damage near the impact site for injured or sham-treated mice (data not shown).

#### Silver staining

**FIG. 2.**
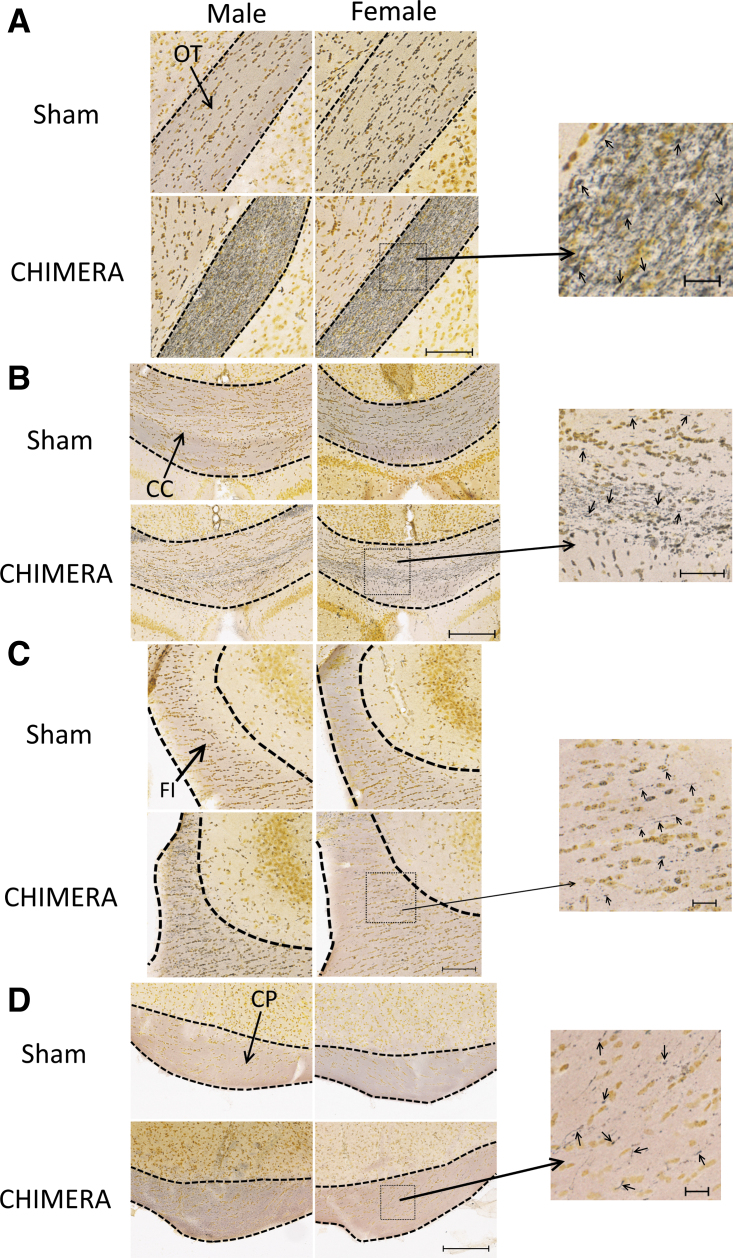
Axonal degeneration as observed by silver staining in the optic tracts (OT; **A**), corpus callosum (CC; **B**), fimbria (FI; **C**), and cerebral peduncles (CP; **D**). Scale bars in (A) and (C) represent 100 μm, (B) and (D) represent 200 μm. Photomicrographs on the right are enlarged images of the boxed regions; scale bars of enlarged regions represent 20 μm (A), (C) and (D), or 50 μm (B). Arrows in enlarged regions point to silver-stained punctate or argyrophilic fibers. All injured mice showed prominent silver staining in the OTs (A), and readily observable silver staining in the CC (B, with enlarged region). About one-third of mice (both male and female) had sparse silver staining in the FI, lateral to CA2, observable with higher magnification (C, enlarged region). Most injured animals also showed white matter damage in the CPs, which was only readily observable at higher magnification (D, enlarged region).

Figure 2 shows axonal damage as qualitatively assessed by silver staining in the OT, CC, FI, and CP. All injured mice had prominent axonal injury in the OTs (Fig. 2A), and readily observable silver uptake indicating axonal varicosities in the CC (Fig. 2B). In the FI, two mice of each sex (out of six) showed very sparse silver uptake, observable on higher magnification (Fig. 2C). In the CP (Fig. 2D), four of five male injured mice and five of six female mice had positive silver uptake, which was only observed on higher magnification.

#### GFAP

Analysis of astrogliosis in the cortex found no effect of Injury, Sex, Side or interactions between those factors in the smCTX near the injury site (F_1,20_ ≤ 1.68, *p* ≥ 0.2099) or in the pCTX (F_1,20_ ≤ 3.95, *p* ≥ 0.0606; data not shown).

In the HP, a four-way ANOVA (Injury × Sex × Region × Side) revealed a main effect of Region on the percent area stained (DG > CA1 = CA2/3; adjusted *p* < 0.0001, *d* ≥ 2.08), but there were no effects of Sex, Injury, or Side, or interactions between these factors or between these factors with Region (F ≤ 3.28, *p* ≥ 0.0513; data not shown). In the FI of the HP, there were no effects of Injury, Sex, Side or interaction among these factors on GFAP staining (F ≤ 1.27, *p* ≥ 0.2730; data not shown).

**FIG. 3.**
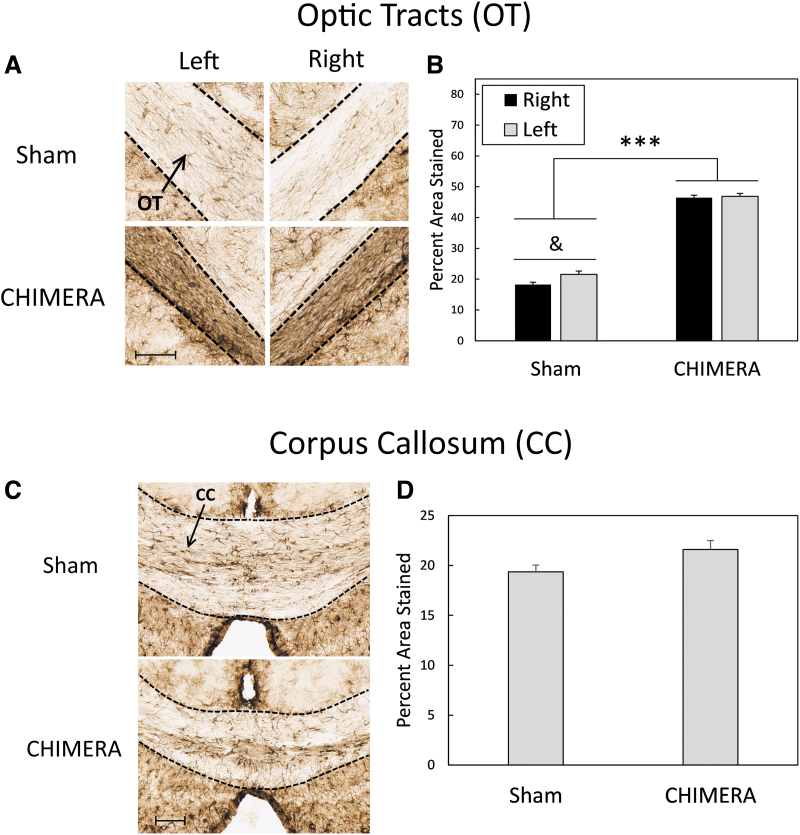
Astrogliosis as measured by GFAP staining in the OTs (**A** and **B**) and CC (**C** and **D**). Sections shown represent the approximate mean of each group, and scale bars represent 200 μm. Data are collapsed by Sex as there were no statistically significant effects of this factor. Closed-Head Impact Model of Engineered Rotational Acceleration (CHIMERA) injuries significantly increased GFAP staining in the OTs on both the left and right sides of the brain (A and B). In sham-treated mice, the left OT had increased staining compared with the right OT. In the CC, the effect of Injury neared significance with injured mice having more GFAP staining than sham-treated animals (C). Asterisks (***) in (B) represent a significant main effect of Injury (CHIMERA > Sham); *p* < .0001. The ampersand (&) in (B) represents an effect of Side in sham-treated mice only (Left > Right); *p* < 0.05. GFAP, glial fibrillary acidic protein; OT, optic tract; CC, corpus callosum.

In the OTs (Fig. 3A, 3B), there was a significant interaction between Side and Injury on levels of GFAP staining (F_1,20_ = 4.38, *p* = 0.0494). Bonferroni-corrected *t*-tests showed significant differences between sham-treated mice and injured mice in both the left and right optic tracts (CHIMERA > Sham; *p* < 0.0001, *d* = 7.25 and 10.15 for left and right sides, respectively); GFAP staining in the left and right optic tracts was equal in injured mice (*p* = 1.0), but in sham-treated mice staining was greater in the left optic tract than in the right optic tract (*p* = 0.0212, *d* = 0.36). The effect of Injury on astrogliosis in the corpus callosum neared significance (F_1,20_ = 3.80, *p* = 0.065, *d* = 0.83; Fig 3C, 3D).

#### Parvalbumin

**FIG. 4.**
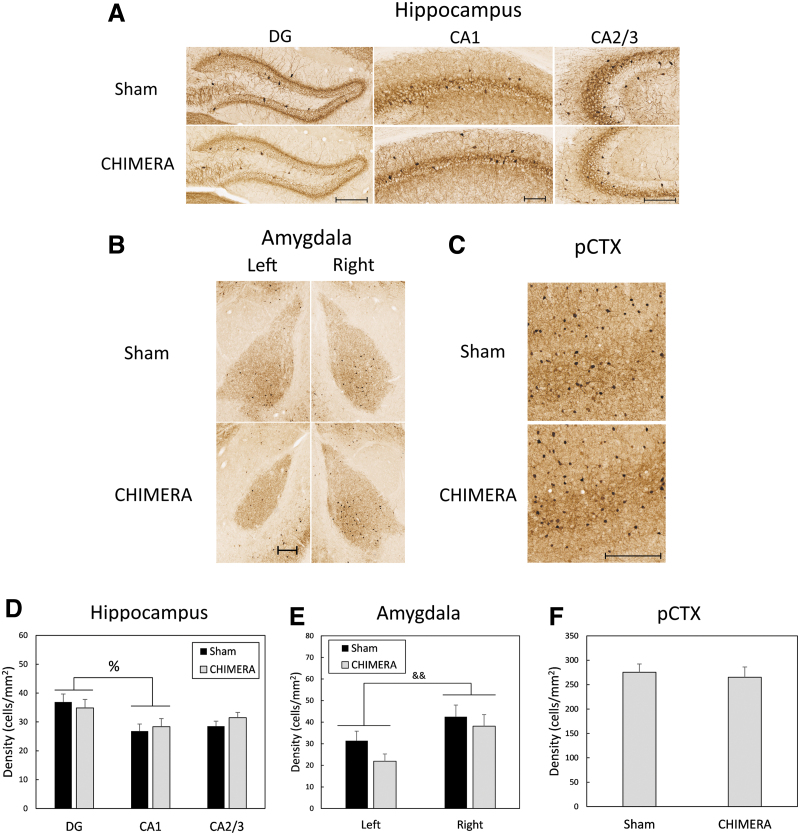
Density of parvalbumin-immunoreactive (PV-IR) cells in the HP **(A, D),** AMY **(B, E),** and pCTX **(C, F).** Scale bars for each region represent 200 μm and photomicrographs represent the approximate mean for each group. There were no effects of Sex on PV-IV density in any regions and data are collapsed by this factor. Data in the HP (A) were analyzed separately for each region (DG, CA1, CA2/3). There was a main effect of Region on cell density in the hippocampus, with greater density of cells in the DG than in CA1 (A, D). In the amygdala, there was a main effect of Side, with a greater density of PV-IR cells in the right amygdala than in the left amygdala (B, E). There were no effects of any factors on cell density in the pCTX (C, F). The percent sign (%) in (D) represents an effect of region (DG > CA1), *p* < 0.05; Ampersands (&&) in (E) indicates a main effect of side, Right > Left, *p* < 0.01. PV-IV, parvalbumin-immunoreactive; HP, hippocampus; AMY, amygdala; pCTX, parietal cortex; DG, dentate gyrus.

**FIG. 5.**
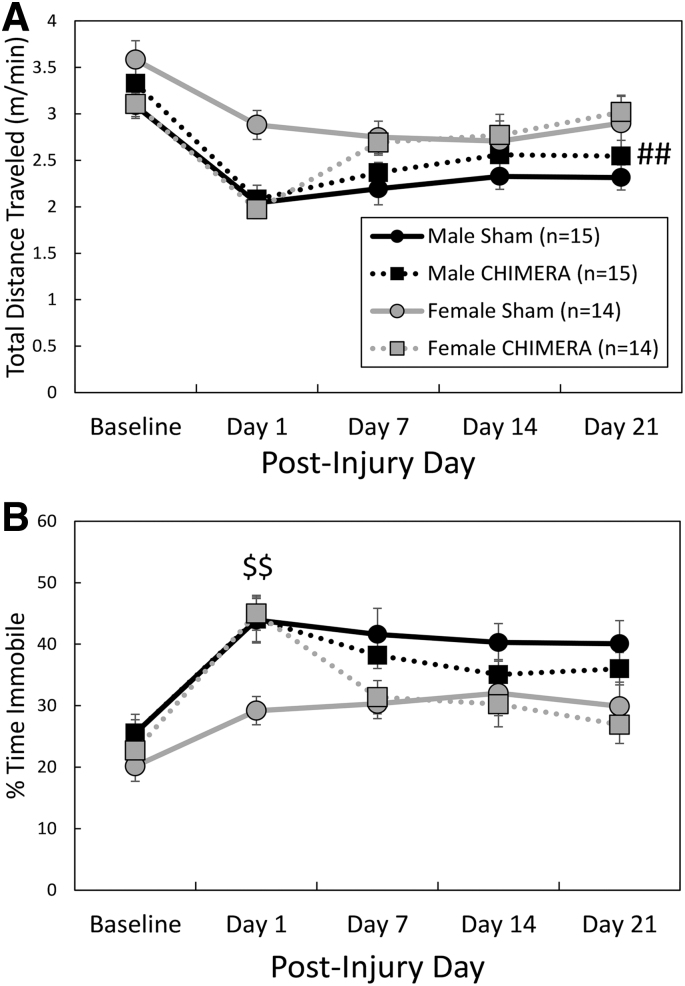
Spontaneous behavior in an open field environment. Legend in **(A)** applies to both panels. All female mice were more active than males as measured by total distance traveled (A). Female injured mice spent significantly more time immobile than female sham controls on Day 1 after the final injury **(B).** The pound signs (##) in (A) represent a main effect of Sex, Female > Male, *p* < 0.01, and the dollar signs ($$) in (B) represent an effect of Injury in female mice only on the indicated day, Female Sham > Female Closed-Head Impact Model of Engineered Rotational Acceleration (CHIMERA), *p* < 0.01.

Figure 4A and 4D shows parvalbumin immunoreactivity in the HP. A four-way ANOVA (Injury × Sex × Region × Side) for PV-IR density in the HP revealed a main effect of Region (F_2,19_ = 4.14, *p* = 0.032). Bonferroni-corrected *t*-tests determined that the density of PV-IR cells was greater in the DG than in CA1 (*p* = 0.012, *d* = 0.60). There were no effects of Injury, Sex, Side, or interactions between those factors with Region (F ≤ 3.67, *p* ≥ 0.070). Representative sections of PV-IR in the right AMY are shown in Fig. 4B. There was a main effect of Side on density of PV cells (F_1,19.8_ = 9.31, *p* = 0.0064, *d* = 0.85, with the right side having a greater density of cells than the left side (right: mean = 40.33, SD = 18.43; left: mean = 26.43, SD = 13.85). There were no effects of Injury or Sex, or interaction effects between the three factors (F_1,19.8_ ≤ 3.24, *p* ≥ 0.0871; Fig. 4E). There were no effects of Injury, Sex, Side, or interactions between the factors on density of PV-IR cells in the pCTX (F_1,20_ ≤ 1.32, *p* ≥ 0.265; Fig. 5C and 5F) or the smCTX (F_1,20_ ≤ 2.99, *p* ≥ 0.0993; data not shown).

### Behavioral results

#### Experiment 1—Spontaneous activity (OF), rotarod, y-maze, Morris water maze

There was an Injury by Day interaction effect on total distance traveled in the OF arena (F_4,215_ = 4.67, *p* = .0012; Fig. 5A); planned contrasts did not reveal significant differences between injured and sham groups on any specific testing days, although hypoactivity of CHIMERA-treated mice on Day 1 following injuries neared significance (adjusted *p* = .0525, *d* = 0.72). There was also a significant main effect of Sex; female mice ambulated greater distances than male mice (F_1,67.4_ = 8.16, *p* = 0.0057, *d* = 0.47). Sham-treated female mice also ambulated at greater speeds than sham-treated males (injury by sex interaction effect: F_1,270_ = 5.56, adjusted *p* = 0.008, *d* = 1.00; data not shown). For time spent immobile during the test session (Fig. 5B), there was a significant three-way (Injury × Sex × Day) interaction effect (F_4,217_ = 2.60, *p* = 0.0371); separate two-way ANOVAs were performed for each Sex. There was no main effect of Injury or Injury by Day interaction effect in males (F < 0.90, *p* > 0.3499). In females, there was a significant Injury by Day interaction effect (F_4,106_ = 9.04, *p* < 0.0001); CHIMERA-treated females spent more time in an immobile state than sham females on Day 1 following injuries (adjusted *p* = 0.0010, *d* = 1.68).

**FIG. 6.**
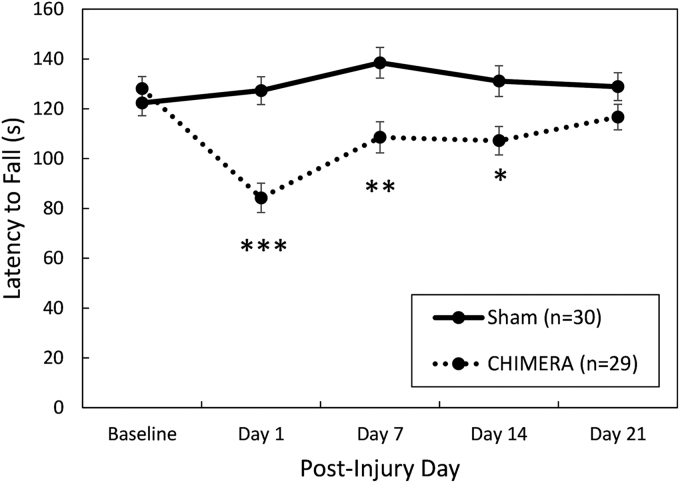
Performance on the rotarod test of motor coordination. There were no statistically significant effects of Sex; data are collapsed by this factor. Closed-Head Impact Model of Engineered Rotational Acceleration (CHIMERA)–injured mice were significantly impaired up to 2 weeks following injury, but on Day 21 their performance was equal to sham-treated controls. Asterisks (*) represent a main effect of Injury on the indicated Day, Sham > CHIMERA: ***p < 0.001; **p < 0.01, *p < 0.05.

On the rotarod test of motor ability (Fig. 6), there was a significant interaction effect between Day and Injury (F_4,220_ = 11.99, *p* < 0.0001). Bonferroni-corrected planned contrasts showed that injured mice fell from the accelerating rod at an earlier time than sham-treated mice on post-injury Days 1 (*p* < 0.0001, *d* = 1.37), 7 (*p* = 0.0020, *d* = 0.88), and 14 (*p* = 0.0195, *d* = 0.73), but by the 3rd week after the injuries the groups had equal performance (*p* = 0.6670).

**FIG. 7.**
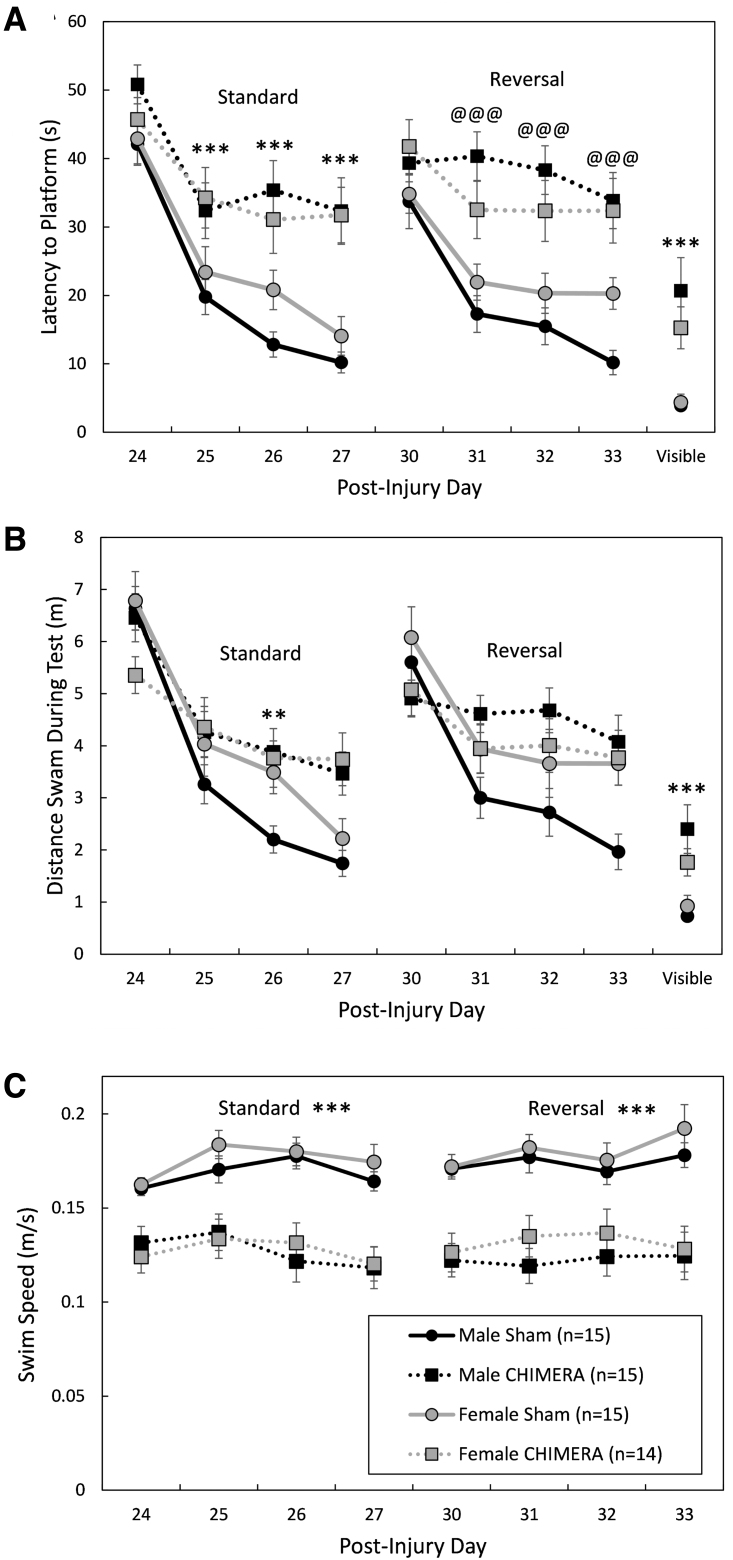
Performance during learning trials in the Morris water maze. Legend in **(C)** applies to all panels. Injured mice were impaired during both standard and reversal learning as measured by the latency to find the hidden platform **(A).** There was a significant main effect of Injury on the latency to locate the platform during the standard trials; during reversal training trials, Closed-Head Impact Model of Engineered Rotational Acceleration (CHIMERA) only had an effect in male mice. When distance swam was analyzed **(B),** the effect of injury was limited to the 3rd standard training day (post-injury Day 26). Swim speed (C) was significantly affected by Injury during both standard and reversal training trials. All injured mice were impaired on the visible platform trials (A and B). Asterisks (*) in (A) and (B) represent an effect of Injury on the given day/trial; in (C) represent a main effect of Injury during standard and reversal trials, CHIMERA < Sham: ***p < 0.001; **p < 0.01. The at symbol (@) in (A) indicates an effect of Injury in male mice only on the represented day, Male CHIMERA > Male Sham; @@@*p* < 0.001.

**FIG. 8.**
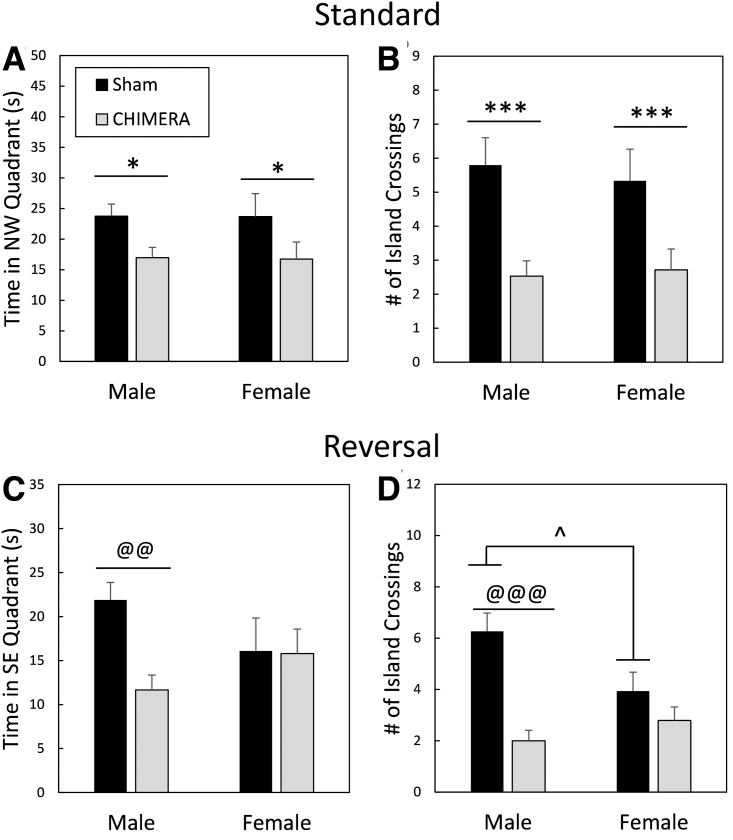
Performance during probe trials in the Morris water maze. Legend in **(A)** applies to all panels. All injured mice had memory impairments during the probe trial following standard training trials as measured by the amount of time spent in the quadrant that formerly housed the platform (northwest [NW]; **A**) and by the number of times they crossed the exact location in which the platform had previously been **(B).** After reversal trials, only male mice were impaired following Closed-Head Impact Model of Engineered Rotational Acceleration (CHIMERA; **C** and **D**). In addition, there was a difference between male and female sham-treated mice in the number of times they crossed the island location (D), with males having superior performance. Asterisks (*) in (A) and (B) represent a main effect of injury, Sham > CHIMERA: ***p < 0.001, *p < 0.05. The at symbol (@) in (C) and (D) represents an effect of Injury in male mice only, Male Sham > Male CHIMERA: @@@*p* < 0.001, @@*p* < 0.01. The caret (^) in (D) indicates a sex difference in sham treated mice, Sham Male > Sham Female: ^*p* < 0.05.

The effects of 4X CHIMERA on cognitive behaviors were test specific. There were no significant effects of Injury or Sex, or interactions between the factors, on the y-maze test of spontaneous alternation behavior (F_1,55_ ≤ 0.576, *p* ≥ 0.451; data not shown). Performance on the Morris water maze (MWM) was significantly impaired by 4X CHIMERA (Fig. 7 and Fig. 8). There was a significant Injury × Day interaction effect on the latency to find the platform on post-injury Days 24-27 (standard spatial training; F_3,165_ = 14.30, *p* < 0.0001; Fig. 7A). Bonferroni-corrected planned contrasts comparing 4X CHIMERA and sham-treated mice on each training day showed that injured mice had longer latencies to find the platform than sham-treated mice on all training days except the first (*p* < 0.0001, *d* = 0.80, 1.16, 1.55 for training Days 2, 3, and 4, respectively). There was also an Injury × Day interaction effect on the distance swam to the platform (or during the maximum 60 sec trial) on Days 24-27 (F_3,163_ = 5.53, *p* = 0.0012; Fig. 7B). Planned contrasts showed that injured mice swam greater distances than uninjured mice on the 3rd training day (post-injury Day 26; adjusted *p* = 0.0012, *d* = 1.08).

During reversal training trials on post-injury Days 31-34, there was a Sex × Injury × Day interaction effect on the latency to find the platform (F_3,167_ = 2.67, *p* = 0.0491; Fig. 7A). Separate two-way ANOVAs (Injury × Day) were performed for each Sex; in females the main effect of Injury neared significance, (F_1,34.4_ = 3.72, *p* = 0.0621) and there was no Injury × Day interaction (F_3,83.4_ = 0.56, *p* = 0.6416) but there was an Injury × Day interaction effect in males (F_3,82.6_ = 7.59, *p* = 0.0002). Planned contrasts showed that male mice that had sustained 4X CHIMERA had longer latencies to find the platform on the 2nd, 3rd, and 4th days of reversal training (adjusted *p* < .0001, *d* = 1.91, 1.86 and 1.93 for Days 2, 3, and 4, respectively). For distance swam during the reversal trials (Fig. 7B), the Injury × Sex interaction effect neared significance (F_1,62.7_ = 3.94, *p* = 0.0514), with males swimming greater distances to the platform (or during the 60 sec test) than females. Although there was a significant Injury × Day interaction effect (F_3,162_ = 5.25, *p* = 0.0017), Bonferroni-corrected contrasts did not reveal significant differences between injured and sham-treated mice on any specific days.

Swim speed of mice was affected by repeated CHIMERA injury (Fig. 7C). There was a main effect of Injury on swim speed during standard spatial training trials (F_1,64.8_ = 46.81, *p* < 0.0001); all injured mice had significantly slower swim speeds than sham-treated mice (*d* = 1.41). During reversal training trials, there was an Injury × Day interaction effect on swim speed (F_3,163_ = 3.14, *p* = 0.0270). Bonferroni-corrected contrasts showed, however, that injured mice had slower swimming speeds on all testing days (*p* < 0.0001; *d* = 1.58, 1.41, 1.13 and 1.32 for Days 1, 2, 3, and 4, respectively). There were no effects of Sex or interaction effects between Sex and any other factors on swim speeds during standard (F ≤ 1.20, *p* ≥ 0.3107) or reversal training trials (F ≤ 0.88, *p* ≥ 0.3511).

MWM probe trials indicated spatial memory impairment following 4X CHIMERA (Fig. 8). There was a main effect of Injury on the amount of time spent in the NW quadrant (that previously housed the platform) during the probe trial following standard training (post-injury Day 28; F_1,55_ = 6.63, *p* = 0.0127; Fig. 8A); injured mice spent less time in the correct quadrant (*d* = 0.68). There was also a main effect of Injury on annulus crossings (Fig. 8B), with sham-treated mice crossing the exact location of the platform a greater number of times during the probe trial than injured mice (F_1,55_ = 16.39, *p* = 0.0002, *d* = 1.08). For the reversal probe trial (Day 35 post-injury), there was an Injury × Sex interaction effect on the amount of time spent in the SE quadrant that previously held the platform (F_1,55_ = 7.64, *p* = 0.0078; Fig. 8C). Planned contrasts showed that injured male mice were impaired compared with sham-treated male mice (adjusted *p* = 0.0012, *d* = 1.34), but there were no differences between female injured and female sham-treated mice (adjusted *p* = 1.0). There were also no differences between male and female injured mice (adjusted *p* = 0.4536) or between male and female sham-treated mice (adjusted *p* = 0.1364).

There was also an Injury × Sex interaction effect on the number of annulus crossings during the reversal probe trial (F_1,55_ = 6.44, *p* = 0.0140; Fig. 8D). Male injured mice were significantly impaired compared with sham-treated male mice (adjusted *p* < 0.0001, *d* = 1.90), but female mice that had sustained 4X CHIMERA had similar performance to sham-treated female mice (adjusted *p* = 0.7840). There were no differences between injured male and female mice (adjusted *p* = 1.0), but sham-treated male mice crossed the annulus a greater number of times than sham-treated females (adjusted *p* = 0.036, *d* = 0.83).

Brain injuries impaired performance on the MWM visible platform trials that were performed on Day 37 post-injury. There was a main effect of Injury on performance (F_1,55_ = 40.59, *p* < 0.0001), with all injured mice taking a greater amount of time to reach the visible platform (*d* = 1.22; Fig. 7A). Male and female injured mice also swam greater distances to the visible platform than sham-treated mice (main effect of Injury: F_1,55_ = 32.20, *p* < 0.0001, *d* = 1.12; Fig. 7B).

#### Experiment 2—Trace fear conditioning

##### Training/association

**FIG. 9.**
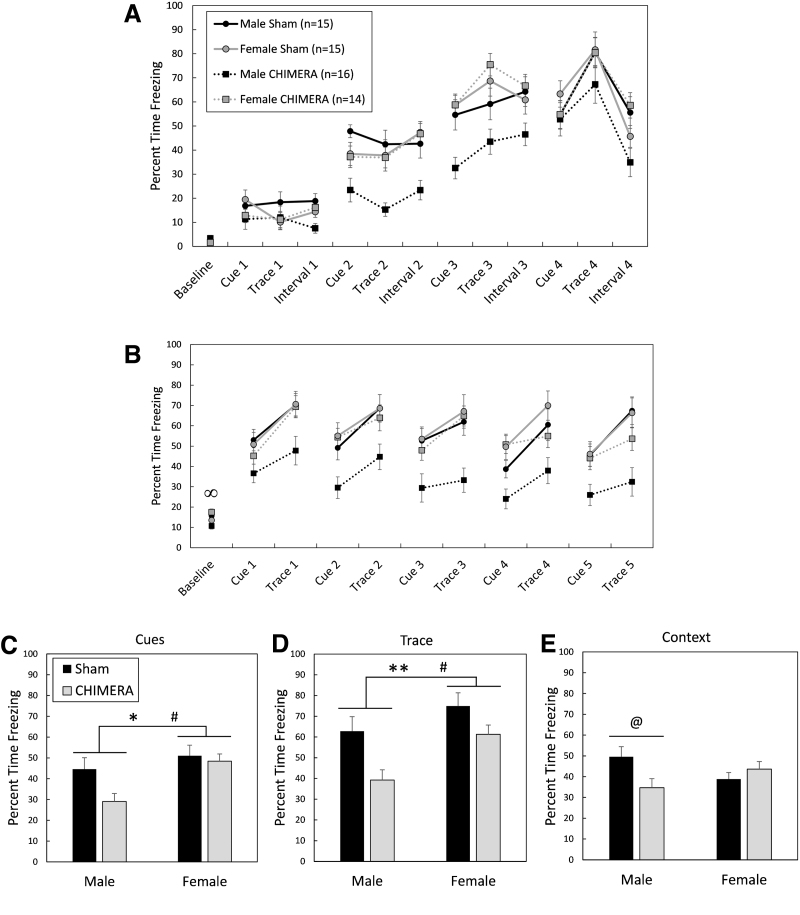
Trace fear conditioning, 24-26 days following 4X Closed-Head Impact Model of Engineered Rotational Acceleration (CHIMERA) or sham procedures. Legend in **(A)** applies to (A) and **(B);** legend in **(C)** applies to (C), **(D),** and **(E).** All mice learned to associate the auditory cue and trace period with a foot-shock (A). During the cue test (B), there was an Injury × Sex interaction effect on the amount of time freezing during the 3-min baseline period, with injured female mice freezing more than injured male mice. The total amount of time freezing during cues (C) and trace periods (D) was affected by both Sex and Injury. Overall, female mice froze more than male mice, and sham-treated mice froze more than injured mice. During the context test (Day 26; E), injury only affected male mice, with injured male mice freezing less than sham-treated male mice. The infinity symbol (∞) in (B) represented an effect of Sex in injured mice only, Female CHIMERA > Male CHIMERA: ∞, p < .05. Asterisks (*) in (C) and (D) indicate an effect of Injury, Sham > CHIMERA: **p < 0.01, *p < 0.05. The pound sign (#) in (C) and (D) represents a main effect of Sex, Female > Male: #*p* < 0.05. The at symbol (@) in (E) represents an effect of Injury in male mice only, Male Sham > Male CHIMERA: @*p* < 0.05.

Brain injury induced by repeated CHIMERA also affected cognitive performance as assessed by TFC in separate mice on Days 24-26 following the final injury (Fig. 9). There were no significant main effects of Injury, Sex, or Injury × Sex interaction effects on freezing behavior during the 3 min baseline period during the training/association period (F_1,65_ < 3.59, *p* > 0.0624; Fig. 9A). Mice learned to associate the auditory cue and the trace/delay period with the subsequent shock, as measured by an increase in freezing behavior over the course of the training period. There was a main effect of Time period on the amount of freezing during the auditory cue (F_1,65_ = 149.38, *p* < 0.0001) and trace (F_1,65_ = 268.58, *p* < 0.0001), with the percent time freezing increasing from the first auditory cue/trace to the final auditory cue/trace in all mice (cue: *d* = 2.05; trace: *d* = 2.85). There were no effects of Injury, Sex, or interactions of those factors with Time period on freezing behavior during auditory cues (F_1,65_ < 1.84, *p* > 0.1798) or trace periods (F_1,65_ < 1.77, *p* > 0.1877) in the training period on Day 24.

##### Cue test

Figure 9B shows behavior of mice during the cue test 25 days following the final injury or sham procedure. There was an Injury × Sex interaction effect on baseline activity in mice during the cue test (F_1,65_ = 6.69, *p* = 0.0119). Planned contrasts showed that injured female mice had greater freezing durations than injured male mice during the baseline period (adjusted *p* = 0.0188, *d* = 0.98). Sham-treated male and female mice had similar freezing durations (adjusted *p* = 1.0) and there were no differences between injured and sham-treated mice of the same sex (adjusted *p* > 0.2488).

An Injury by Sex interaction effect on total freezing behavior during auditory cues neared significance (F_1,65_ = 3.54, *p* = 0.0644), and there were main effects of Injury (F_1,65_ = 6.17, *p* = 0.0155) and Sex (F_1,65_ = 6.96, *p* = 0.0104) on total freezing behavior during auditory cues (Fig. 9C). Injured mice froze less than sham-treated mice (*d* = 0.49), and male mice froze less than female mice (*d* = 0.64). The Injury × Sex interaction effect also neared significance for total freezing behavior during the trace periods (F_1,65_ = 3.14, *p* = 0.0810; Fig. 9D), and there were significant main effects of Injury (F_1,65_ = 9.56, *p* = 0.0029) and Sex (F_1,65_ = 5.18, *p* = 0.0261). Injured mice were impaired and showed less freezing than sham-controls during trace periods (*d* = 0.65), and male mice showed less freezing behavior than female mice (*d* = 0.53).

##### Context test

There was an Injury × Sex interaction effect on freezing behavior during the context test in mice tested on Day 26 post-injury (F_1,65_ = 6.96, *p* = 0.0104; Fig. 9E). Bonferroni-corrected planned contrasts showed that CHIMERA-injured male mice were impaired compared with sham-treated male mice, showing less freezing behavior (*p* = 0.0384, *d* = 0.84), but injured and sham-treated female mice had equal performance (*p* = 1.0). Injured and sham-treated mice of the opposite sex also had similar performance (*p* = 0.3224 and 0.2212, respectively).

## Discussion

Table 1 summarizes the behavioral findings in this study.

**Table 1.** Summary of Behavioral Responses following Repeated CHIMERA

Response dimension	Injury effects	Main effect of Sex or Injury × Sex Effect
Righting reflex (Fig. 1B)	Significant effect of Injury (Injury > Sham) on all injury days	No effect
OF total distance traveled (Fig. 5A)	Injury × Day interaction. Hypoactivity in injured mice neared significance (adjusted *p* = 0.0525) on post-injury Day 1.	Main effect of sex (Female > Male)
OF speed while mobile (not shown)	No main effect	Injury × Sex interaction. Female Sham speed > Male Sham speed
OF time immobile (Fig. 5B)	Effect of injury in females only.	Injury × Day interaction in females; on Day 1, injured females were more immobile than sham-treated females
Rotarod (Fig. 6)	Injury × Day interaction. Injured mice were impaired on Days 1, 7, and 14, but not on Day 21 following injuries.	No effect
Y-maze spontaneous alternation (not shown)	No effect	No effect
MWM latency—standard training (Fig. 7A)	Significant Injury × Day interaction. Injured mice had significantly longer latencies to find the platform on Days 2, 3, and 4 of training.	No effect
MWM distance—standard training (Fig. 7B)	Significant Injury × Day interaction. Injured mice had significantly longer distances to find the platform on Day 3 of training.	No effect
MWM latency—reversal training (Fig. 7A)	Effect of injury in males only.	Injury × Day effect in males only; injured male mice had significantly longer latencies to find the platform than sham male mice on Days 2, 3, and 4 of reversal training
MWM distance—reversal training (Fig. 7B)	No effect	No effect
MWM swim speed (Fig. 8C)	Main effect of Injury (Sham > CHIMERA)	No effect
MWM probe trial—standard (Fig. 8A and 8B)	Main effect of Injury on time spent in correct (NW) quadrant and number of annulus crossings (Sham > CHIMERA)	No effect
MWM probe trial—reversal (Fig. 8C and 8D)	Effect of Injury in males only	Time in correct (SE) quadrant; Male Sham > Male CHIMERA. Number of annulus crossings; Male Sham > Male CHIMERA. There was also a significant difference between sham-treated mice of the opposite sex, with male sham mice crossing the annulus a greater number of times than female sham mice.
MWM visible platform trials (Fig. 7A and 7B)	Main effect of injury on latency and distance to platform; CHIMERA > Sham	No effect
TFC—training baseline (Fig. 9A)	No effect	No effect
TFC—cue test baseline (Fig. 9B)	No effect	Injured female > Injured male
TFC—cue test, total freezing during cues (Fig. 9C)	Main effect of injury, CHIMERA < Sham	Main effect of sex, Male < Female
TFC—cue test, total freezing during trace periods (Fig. 9D)	Main effect of injury, CHIMERA < Sham	Main effect of sex, Male < Female
TFC—context test (Fig. 9E)	Effect of Injury in males only	Male CHIMERA < Male Sham

CHIMERA, Closed-Head Impact Model of Engineered Rotational Acceleration; OF, open field; MWM, Morris water maze; TFC, trace fear conditioning.

### Summary of pathological findings

Repeated CHIMERA at an impact energy of 0.7 J resulted in rare episodes of apnea, followed by loss of consciousness as measured by the duration to the return of the righting reflex, as found previously following CHIMERA injuries.^53^ Pathologically, the repeated injury caused diffuse axonal injury evidenced by increased astrogliosis in the optic tracts and silver uptake in the optic tracts (OT), corpus callosum (CC), and cerebral peduncles (CP) of the brainstem. Axonal damage in the CC and OT has been reported extensively following single or repeated CHIMERA with silver staining, GFAP, Iba1, amyloid precursor protein, myelin basic protein, and neurofilament,^37^ and has been suggested to be indicative of a diffuse injury pattern reflective of coup and contrecoup injuries, respectively.^14^

No changes were found in any brain regions in the density of GABAergic parvalbumin (PV)-expressing interneurons. The HP and AMY contain a large proportion of PV interneurons, which are intricately involved in neuronal circuits for memory.^54–57^ Decreases in PV immunoreactivity have been reported in the HP following single or repetitive focal concussive^39,58^ or lateral fluid percussion injury,^59–62^ which results in a mixed focal-diffuse injury. In addition, CCI resulted in a reduction in PV-expressing interneurons in both the HP and AMY.^63^ It is likely that the lack of changes in PV-expressing neurons in gray matter in the current study is due to the milder, diffuse nature of the CHIMERA injuries.

Pathological findings from previous CHIMERA experiments with impact energies up to 0.7 J have been limited to white matter,^14,53^ with no gray matter damage, as found in the current study. Bashir and colleagues demonstrated that increasing the impact energy to 2.5 J and employing an interface between the piston and skull to dissipate the impact force across a greater area of the skull, thus avoiding skull fractures, resulted in increased inflammation in cortical regions but not in the CA1 area of the hippocampus after a single injury.^64^ However, Sauerbeck and colleagues, also employing an interface between the piston and skull (modCHIMERA), reported increased microgliosis (Iba1 staining) in the hippocampus following a single impact at 2.1 J.^43^ Taken together, these studies demonstrate that, similar to clinical mTBI, white matter is particularly sensitive to the rotational-acceleration injury induced by the CHIMERA model and higher impact energies are required to produce gray matter pathology.

Although the pathology reported here is limited, there was no evidence of sex differences in the diffuse white matter injury following repeated CHIMERA. Accumulation of β-APP has been demonstrated to be similar in male and female mice following modCHIMERA in the CC (1.7 or 2.1 J), but there was a sex difference in the FI, with significantly reduced β-APP in injured female mice following a single 2.1 J modCHIMERA impact compared with injured male mice.^43^ Here, no injury or sex differences were found in astrogliosis (GFAP staining) in the FI following 4X CHIMERA (0.7 J), although axonal pathology that could have been revealed with other biomarkers cannot be ruled out. Sex differences in white matter astrogliosis have also been reported in rodent models of focal repetitive concussive brain injury (CBI), with female animals having reduced inflammation compared with males,^13,39^ although other studies report no sex differences.^46,65^

### Morris water maze (MWM) deficits in CHIMERA-injured mice

The data in this study confirm multiple previous publications demonstrating hippocampal-dependent spatial cognitive deficits following single or repetitive CHIMERA injuries.^14,43,66^ The Morris water maze is among the most widely employed behavioral tests for assessing functional deficits following all methods of experimental TBI,^17^ and brain-injured mice in this study demonstrated impairments on this test during both training (learning) trials and probe (memory) trials following multiple CHIMERA injuries. The normal spontaneous ambulatory behavior in the OF and resolved rotarod deficits by 3 weeks post-injury suggested that motor impairments would not interfere with performance in the MWM. However, both male and female injured mice had substantially slower swim speeds compared with sham controls, implicating motor impairments as a confound in the interpretation of MWM data.

Increased silver staining was observed in the CP of the brainstem in many animals. As corticospinal fibers descending to the spinal cord travel through the CP, the axonal damage observed in this region may contribute to the motor deficits found during rotarod and MWM testing. This description of brainstem pathology is limited and should be considered preliminary. However, brainstem axonopathy, specifically in the corticospinal tract, has been demonstrated using the CLARITY method in an alternate model of impact-acceleration injury in mice, and the axonal lesions were associated with atrophy of corticospinal neurons.^67^ Also of important consideration is the integrity of the mesencephalic locomotor region circuitry, which can initiate and control coordinated locomotor activities such as swimming via central pattern generators in the spinal cord.^68,69^

Using “distance to platform” rather than “latency to platform” can partially circumvent analysis problems and conclusions when there are swim speed differences. When “distance to platform” data were analyzed, there were significant differences between injured and sham groups on only one training day (standard training Day 3). This finding suggests that injured mice may have still learned the location of the platform but were not able to swim there as quickly as control mice due to motor deficits. However, injured mice also showed greater distances (and latencies) to the platform during visible platform trials, suggesting there may also be motivational and/or visual deficits contributing to poor performance. Poorer performance on visible platform trials following multiple CHIMERA injuries has been reported in another recent study, which also found impaired performance on a visual cliff task and reduced visual evoked potentials, providing further evidence for behavioral and physiological visual dysfunction.^38^ Like many other murine models of CBI, with or without rotational acceleration,^13,39–42,46,70-72^ pathological analysis of mice following CHIMERA (including the current study) finds significant and often prolonged increased astrogliosis and/or microgliosis in the optic tracts as assessed by GFAP or Iba1 staining, respectively.^14,38,66^ Decreased performance on the optokinetic behavioral response test, coupled with decreases in the number of retinal ganglion cells, has also been reported following single or repeated concussive TBI.^73^

### Injury-induced cognitive dysfunction in the fear conditioning paradigm

Despite indications from MWM testing that brain-injured mice suffered hippocampal-dependent learning and memory deficits, these conclusions were clouded by evidence that the injured mice also had visual and motor deficits that would have interfered with their ability to perform the task. Thus, a separate group of animals were tested, at the same time-point post-injury as the animals tested in the MWM, in the hippocampal-dependent TFC paradigm, which is less dependent on motor and visual function. Tactile, auditory, and olfactory cues play a significant role in triggering memories, and performance is based on freezing behavior, rather than active movement. All mice, injured and sham, learned to associate a white noise stimulus and a 20-sec trace period with a subsequent foot-shock, as measured by increased freezing to the auditory stimulus and trace period between the stimulus and shock during the training period. However, all injured mice froze less during the tone and trace periods during the cue test, suggesting impaired memory. In addition, injured male mice showed evidence of impaired memory during the context test. These results support the results of the MWM experiment, which indicated impaired hippocampal-dependent memory function.

The TFC paradigm has been employed rarely following experimental TBI. In a recent study assessing the effects of blast TBI, Weiss and colleagues demonstrated reduced freezing after injury to the cue in a TFC paradigm as well as decreased freezing in the context test, which showed negative correlation with fractional anisotropy values in the CC.^74^ More often reported are results following experimental TBI and delay fear conditioning (DFC), in which the foot shock co-terminates with the auditory cue. The context test in the DFC paradigm is hippocampal-dependent, and contextual FC is often utilized as a specific behavioral test of hippocampal function.^75-77^ Deficits on the DFC context test in male mice have been reported following fluid percussion injury,^76–80^ repetitive CBI,^39,70,75^ and blast neurotrauma.^74^

### Neural underpinnings of cognitive dysfunction following CHIMERA

No pathology was found here to account for the behavioral deficits observed. No inflammation as measured by GFAP staining was found in the HP, nor were there any observable gross H&E differences between injured and sham-treated mice. Also of particular interest was no change in PV-expressing cell density in the AMY and the HP, as PV-IR cells in these regions have been demonstrated to be important for memory consolidation.^54,56^ PV-IR cell density in the dentate gyrus of the HP has been previously reported to decrease following experimental TBI,^39,58,61,62^ and this cell loss has been associated with impaired performance on contextual and cued DFC test performance following repetitive concussive brain injuries in male mice.^39^ But, another pathological mechanism must account for impaired TFC performance following repeated CHIMERA injuries, as well as for the observed MWM deficits.

Overall, neuronal loss, as well as neuronal dysfunction, have been reported in the hippocampus in many pre-clinical rodent models of TBI. Post-TBI changes in hippocampal neuronal function include impaired long-term potentiation, cell excitability changes and slower axon conduction velocities, often which are associated with cognitive deficits.^75,76,81–84^ Recently, Bashir and colleagues performed *ex vivo* CA1 hippocampal field recordings 6 h and 14 days following a single CHIMERA in mice with an interface (2.5 J) and reported decreased peak event amplitudes at both time-points.^64^ The decrease at the acute time-point was associated with a slight reduction in a marker of presynaptic glutamatergic vesicles (vesicular glutamatergic transporter 1), suggesting that acute synaptic loss may contribute to the observed network changes.^64^ Although more studies are needed, these data suggest that alterations in physiological properties may be at least partially responsible for cognitive deficits.

Also of consideration is that both MWM and FC deficits could be considered a result of brain damage more diffuse than an insult to a specific region such as the HP. The diffuse white matter injury, observed in nearly all animal models of mTBI, is a possible mechanism of functional deficits. Also, the pCTX, in addition to the HP, is involved in processing complex spatial information as required during acquisition trials in the MWM, although there are conflicting perspectives on the relative roles of each, discussion of which goes beyond the scope of this article.^85-87^ Similarly, extra-hippocampal damage could explain TFC results. Contextual memories can be spared when the hippocampus is damaged prior to association trials,^88,89^ as possible in the current study. It has been suggested that if the hippocampus is impaired, the neocortex is able to form the contextual representation, though much more slowly than with an intact hippocampus.^90^ Krukowski and colleagues examined properties of layer V prefrontal cortex neurons following repetitive CHIMERA, and found that neurons projecting subcortically had an increase in the frequency of spontaneous excitatory post-synaptic currents.^91^ Thus, there are preliminary results from the CHIMERA model suggesting changes in both hippocampal and cortical cell properties that may contribute to cognitive dysfunction following injury.

### Sex differences in cognitive dysfunction following repeated CHIMERA

Male and female mice had similar motor deficits following repeated CHIMERA as evidenced by equivalent rotarod deficits and reduced swim speeds in the MWM. However, both the MWM probe trials and TFC results suggest that male mice suffered more hippocampal-dependent cognitive deficits than female mice following CHIMERA brain injuries. Sauerbeck and colleagues also described a slight advantage of injured female mice over injured male mice in the MWM following modCHIMERA (1.7 J).^43^ We have previously described sexually dimorphic cognitive dysfunction in the MWM^13^ and in the DFC paradigm,^39^ with male mice having greater cognitive deficits than female mice following repetitive CBI without rotational acceleration; however, some studies have shown no differences between the sexes following injury in active avoidance tasks.^46,92^ Translational TBI studies inclusive of both sexes have been increasing in number over the past several years, and many are showing that female animals have an advantage in behavioral studies over males. In a recent review of 43 studies that examined sex differences in many outcome criteria following FPI, CCI or CBI, injured females outperformed males in 55% of the studies, and males had better outcomes in none of the studies.^93^

The potential underlying causes of the observed sex differences are numerous. It is well known that the sex hormones, estrogen and progesterone, exert neuroprotective effects in animal models of neurotrauma.^94–98^ Experimental approaches to the exploration of sex as a biological variable (SABV) in neurotrauma research were recently reviewed, and include methods such as monitoring the estrous cycle of female animals, performing gonadectomies with and without hormone replacement, and employment of the four core mouse genotype model.^99^ Some of these procedures were performed in valuable earlier studies using classical TBI models such as CCI, FPI, and weight-drop,^100–103^ but are lacking in present experiments in models growing in popularity such as CBI, blast, and CHIMERA. Consideration of SABV, including the effects of sex steroids, their receptors, and other influences such as epigenetics will increase our understanding of the biological underpinnings of behavioral differences between males and females following injury.

### Summary and clinical implications

In summary, this study has demonstrated cognitive deficits on two hippocampal-dependent tasks following repeated CHIMERA brain injuries. Further, the tests indicated male mice showed more cognitive deficits than female mice, which is consistent with the developing literature. The injured subjects' difficulty with swimming in the MWM underscores the importance of ensuring the ability of subjects to adequately perform behavioral assessments and emphasizes the need to sometimes employ more than one test in a specific behavioral domain to support a conclusion regarding functional effects of experimental manipulations. Pathologically, the injury was characterized by diffuse astrogliosis and silver staining in white matter tracts, and lack of observable injury in gray matter. Although preliminary, mild brainstem damage was described qualitatively following repeated CHIMERA injuries, and more pathological assessments are needed in this region.

Clinical studies demonstrating sex differences in cognitive function following repeated brain injuries continue to result in mixed conclusions,^104,105^ and the reasons for this are unclear. There is evidence that women are at greater risk than men for sustaining concussions during contact sports, thus, it is of great importance to continue to be inclusive of both sexes in pre-clinical TBI research. Further, the CHIMERA model provides a commercially available, clinically relevant platform that allows standardization of injury parameters and direct comparison of results across laboratories.

## Supplementary Material

Supplemental data

Supplemental data
