# Supplementary material for: Hippocampal-Dependent Cognitive Dysfunction following Repeated Diffuse Rotational Brain Injury in Male and Female Mice
Source: J Neurotrauma. 2021 May 13;38(11):1585–606. doi: 10.1089/neu.2021.0025 (PMC8126427; doi:10.1089/neu.2021.0025)

**Supplemental Figure 1.** Regions of interest for GFAP- (**A**), parvalbumin (**B**), and silver-stained (**C&D**) sections. The parietal cortex (pCTX) and all regions of the hippocampus (DG, CA1, CA2/3) were analyzed for both astrocytosis (GFAP; **A**) and density of GABAergic interneurons (parvalbumin; **B**). The presence of GFAP was also assessed in the optic tracts (OPT), fimbria (FI), and the corpus callosum (CC), and GABAergic interneuron density is reported in the amygdala (AMY). Silver-stained images were examined for the presence of argyrophilic structures in white matter tracts: OPTs, FI, CC (**C**) and cerebral peduncles (CP) in the brainstem (**D**).


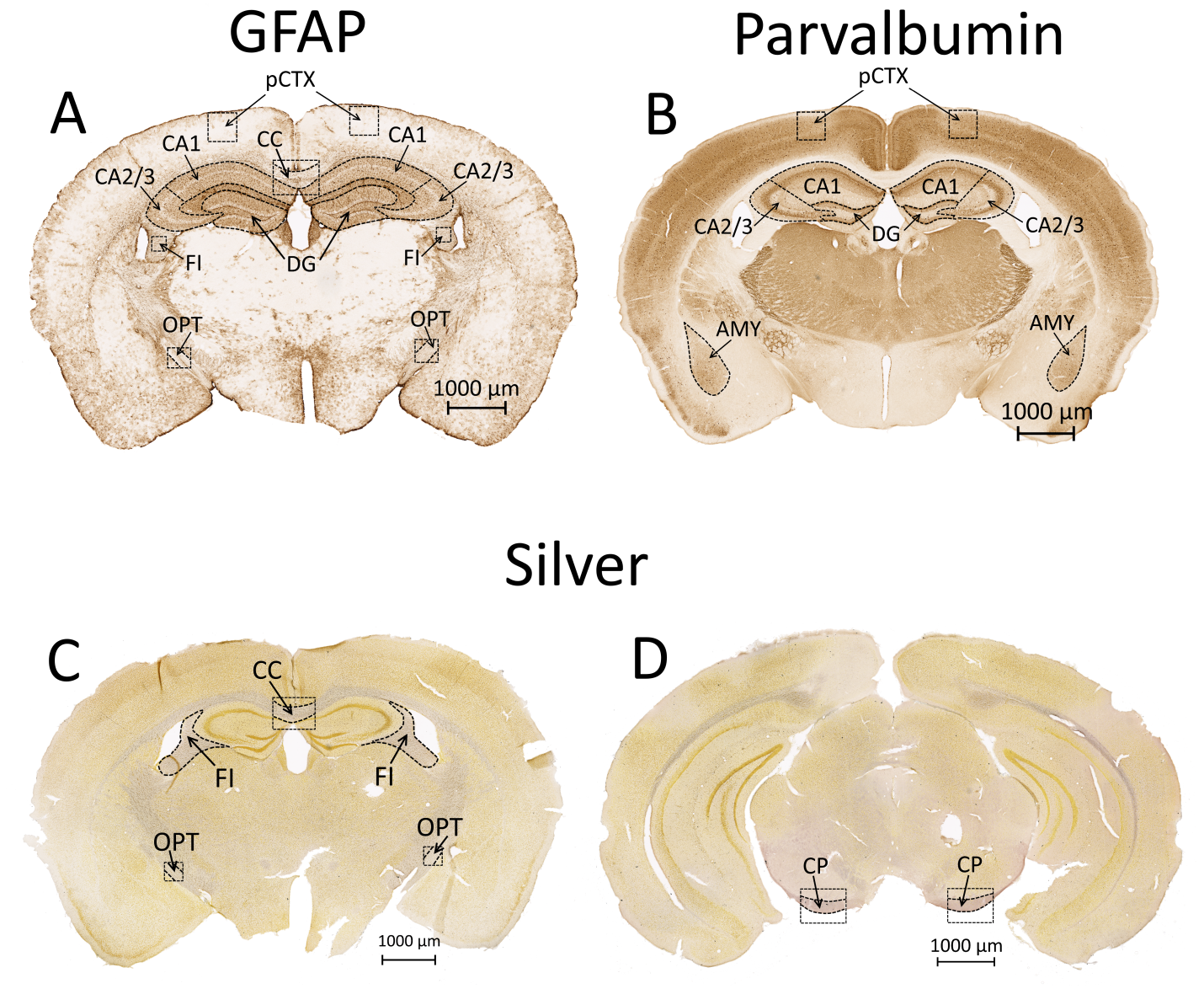

Supplement: Supplemental data [file Supp_FigS1.docx]
