# Supplementary material for: Hippocampal-Dependent Cognitive Dysfunction following Repeated Diffuse Rotational Brain Injury in Male and Female Mice
Source: J Neurotrauma. 2021 May 13;38(11):1585–606. doi: 10.1089/neu.2021.0025 (PMC8126427; doi:10.1089/neu.2021.0025)

**Supplemental Figure 2**. Body weights following CHIMERA injuries. There was a main effect of brain injuries on body weights of both male and female mice on Days 7, 14, 28 and 35 after injuries. Asterisks (***) indicate a main effect of injury, Sham > CHIMERA, *p* < .001.


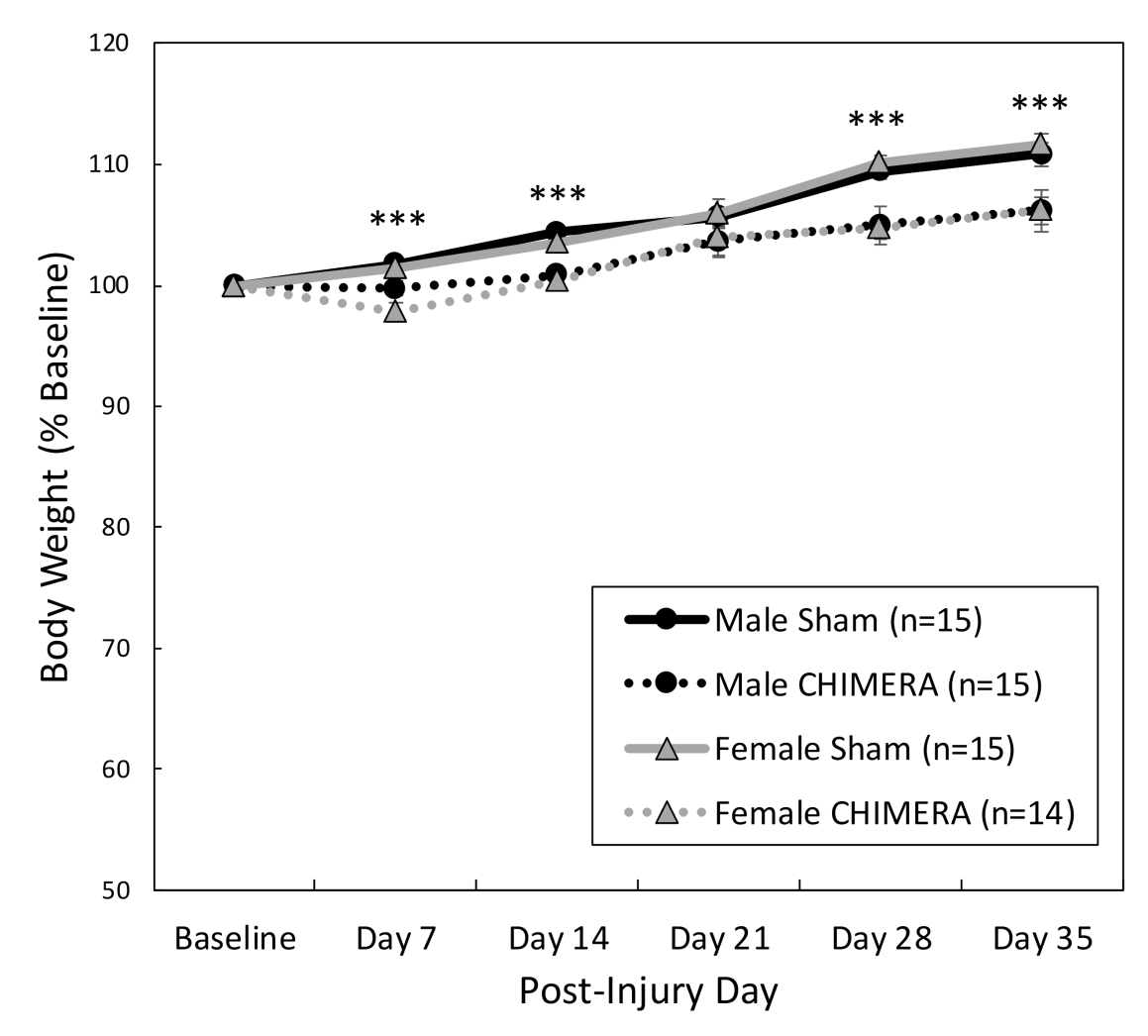

Supplement: Supplemental data [file Supp_FigS2.docx]
